# New lanostane-type triterpene acids from *wolfiporia extensa*

**DOI:** 10.1186/1752-153X-6-39

**Published:** 2012-05-06

**Authors:** Gaimei She, Nailiang Zhu, Shuai Wang, Yang Liu, Yinying Ba, Changqing Sun, Renbing Shi

**Affiliations:** 1 School of Chinese Pharmacy, Beijing University of Chinese Medicine, No. 6, Zhonghuan South Road, Wangjing District, Beijing, 100102, People’s Republic of China

## Abstract

**Backgroud:**

Dried sclerotia of *Wolfiporia extensa* (Polyporaceae) is used to invigorate the spleen and to tranquilize the mind in Chinese herbal medicine. Lanostane-type triterpene acids were regard as major secondary metabolites from dried sclerotia of *W*. *extensa*.

**Results:**

Three new lanostane-type triterpene acids, 3-*epi*-benzoyloxyl-dehydrotumulosic acid (**1**), 3-*epi*-(3′-*O*-methyl malonyloxy)-dehydrotumulosic acid (**2**) and 3-*epi*-(3′-hydroxy-3′-methylglutaryloxyl)-dehydrotumulosic acid (**3**), were isolated from the sclerotia of *W*. *extensa*, together with 3 known lanostane derivatives (**4**–**6**). Their structures were elucidated on the basis of spectroscopic analysis, including 1D and 2D-NMR techniques.

**Conclusion:**

Six lanostane derivatives including three new triterpene acids and three known compounds were reported from the sclerotia of *W*. *extensa* in this paper.

## Background

**Figure 1:**
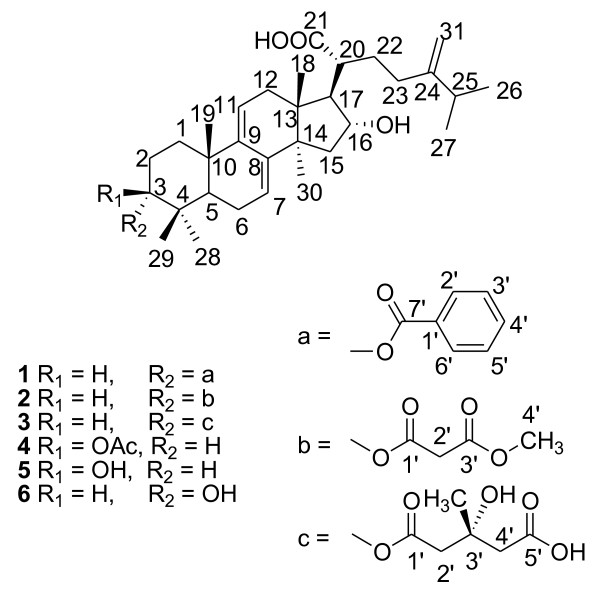
Structure of compounds 1–6.

Dried sclerotia of *Wolfiporia extensa* (Polyporaceae), well known as ‘*Fu-Ling*’ in China, is used to invigorate the spleen and to tranquilize the mind in Chinese herb medicine [1]. In combination with some other herbs, it shows activities as diuretic, sedative and analgesic [2]. Lanostane-type triterpenes were reported as major secondary metabolites, which are characterized with hydroxyl groups at C-16 position, and with a C-21 carboxylic acid group. A number of lanostane-type triterpene acids have been reported from dried sclerotia of *W*. *extensa*, in which some lanostane derivatives showed activities in the anti-tumor, anti-inflammatory and anti-oxidant activities [3-9]. As part of our continuing research on chemical constituents from Traditional Chinese Medicine (TCM) [10-12], three new lanostane-type triterpene acids, 3-*epi*-benzoyloxyl-dehydrotumulosic acid (**1**), 3-*epi*-(3′-*O*-methyl malonyloxy)-dehydrotumulosic acid (**2**) and 3-*epi*-(3′-hydroxy-3′-methylglutaryloxyl)-dehydrotumulosic acid (**3**) were isolated from the dried sclerotia of *W*. *extensa*, together with three known lanostane derivatives (**46**) (Figure 1). Here we report the structure elucidation of the new compounds as follows.

## Results and discussion

The dried sclerotia of *W*. *extensa* were extracted with 95% ethanol as described in Experimental part. The ethanolic extract was concentrated under reduced pressure to small volume and the solution was fractionated with a HPD-826 macroporous adsorptive resin column eluting with H_2_O and 90% EtOH. The 90% EtOH fraction was concentrated and repeatedly fractionated on reverse-phase ODS, and on silica gel column to obtain six lanostane-type triterpene acids (**16**). Of them, **46** were identified as known compounds, dehydropachymic acid (**4**) [7], dehydrotumulosic acid (**5**) [13] and 3-*epi*-dehydrotumulosic acid (**6**) [13] (Figure 1) by spectroscopic methods and comparison with reported data. Compounds **13** were identified as new compounds based on a detailed analysis of NMR as described below (Tables 1 and 2).

**Table 1:** ^**1**^**H-NMR data of 1–3 (at 500 or 600 MHz, in C**_**5**_**D**_**5**_**N;*δ*in ppm,*J*in*Hz*)**

**position**	**1**	**2**	**3**
1	1.73, m 1.82, td (9.6, 3.2)	1.65, m 1.75, dd (13.8, 3.0)	1.69, m 1.88, m
2	1.91, m 1.97, m	1.79, dt (6.6, 3.0) 1.85, d (12.0)	1.78, ddd (15.6, 6.4, 2.8)1.86, m
3	5.09, br s	4.86, br s	4.94, br s
5	1.88, dd (10.0, 4.0)	1.68, t (5.1)	1.76, dd (9.2, 6.4)
6	2.08, m 2.09, m	2.00, m 2.01, m	2.02, m 2.03, m
7	5.64, br s	5.57, br s	5.57, br s
11	5.39, d (5.6)	5.38, d (6.0)	5.39, d (6.0)
12	2.42, dd (15.6, 5.2) 2.66, d (16.8)	2.42, dd (18.0, 6.6) 2.66, d (18.0)	2.42, dd (17.2, 6.8) 2.66, d (16.4)
15	1.95, d (12.4) 2.47, dd (12.8, 9.2)	1.91, d (13.2) 2.45, t (3.9)	1.91, m 2.45, dd (12.4, 8.8)
16	4.52, t (6.8)	4.51, t (7.2)	4.52, t (6.8)
17	2.86, dd (11.2, 5.6)	2.85, dd (11.4, 6.0)	2.84, dd (11.2, 5.6)
18	1.06, s	1.05, s	1.04, s
19	1.04, s	0.99, s	1.00, s
20	2.95, td (10.8, 2.4)	2.94, dd (10.8, 3.0)	2.92, td (10.8, 2.0)
22	2.46, m 2.68, m	2.51, m 2.63, m	2.42, m 2.61, m
23	2.37, m 2.55, br d (11.6)	2.38, m 2.54, m	2.38, m 2.54, m
25	2.29, m	2.29, m	2.27, m
26	0.97, d (6.8)	0.97, d (6.6)	0.97, d (6.8)
27	0.99, d (6.8)	0.98, d (6.6)	0.99, d (6.8)
28	0.92, s	0.87, s	0.90, s
29	0.95, s	0.90, s	0.96, s
30	1.48, s	1.42, s	1.41, s
31	4.84, br s 4.97, br s	4.83, br s 4.97, br s	4.83, br s 4.96, br s
2′	8.18, d (7.2)	3.60, s	3.12, d (15.2) 3.16, d (15.2)
3′	7.35, t (7.6)	–	–
4′	7.46, t (7.4)	3.63, s	3.02, d (14.4) 3.08, d (14.4)
5′	7.35, t (7.6)	–	–
6′	8.18, d (7.2)	–	–
-CH_3_	–	–	1.71, s

**Table 2:** ^**13**^**C-NMR Data of 1–3 (at 125 or 150 MHz, in C**_**5**_**D**_**5**_**N;*δ*in ppm)**

**Position**	**1**	**2**	**3**
1	31.2	30.8	31.1
2	23.5	23.2	23.4
3	79.0	79.6	78.2
4	37.7	36.8	36.7
5	45.3	44.7	44.8
6	23.2	23.1	23.1
7	120.8	120.8	120.7
8	142.9	142.7	142.8
9	146.0	146.0	146.0
10	37.2	37.6	37.6
11	116.7	116.6	116.5
12	36.2	36.2	36.2
13	45.1	45.1	45.1
14	49.5	49.5	49.5
15	44.4	44.4	44.4
16	76.4	76.4	76.4
17	57.6	57.6	57.6
18	17.6	17.6	17.6
19	22.7	22.6	22.7
20	48.5	48.5	48.5
21	178.6	178.7	178.6
22	31.4	31.4	31.4
23	33.2	33.2	33.2
24	156.1	156.0	156.1
25	34.1	34.1	34.1
26	22.0	22.0	22.0
27	21.9	21.8	21.8
28	28.1	27.9	28.1
29	22.4	22.3	22.5
30	26.6	26.6	26.6
31	107.0	107.0	107.2
1′	131.4	167.6	171.4
2′	129.8	41.9	46.3
3′	128.9	166.4	69.9
4′	133.2	52.2	46.4
5′	128.9	–	174.6
6′	129.8	–	–
7′	165.9	–	–
3′-Me	–	–	28.4

Compound **1** was obtained as a colourless crystal in CH_3_OH. The molecular formula was determined as C_38_H_52_O_5_ from its positive HRESI-MS ([M + H]^+^*m*/*z* 589.3864) and ^13^ C-NMR spectrum. The UV spectrum showed absorption at 234 nm, indicating the presence of a Δ^7,9(11)^ diene moiety, which was further supported by an absorption band at 1641 cm^-1^ in the IR spectrum. Strong IR absorption at 3400 and 1710 cm^-1^ indicated the carboxyl group in **1**[13]. The ^1^ H-NMR spectrum of **1** showed signals from two secondary methyls (*δ* 0.97 and 0.99, each 3 H, d, *J* = 6.8 *Hz*), five tertiary methyls (*δ* 0.92, 0.95, 1.04, 1.06 and 1.48, each 3 H, s), two oxygen-bearing methylenes *δ* 4.52 (1 H, t, *J* = 6.8 *Hz*) and *δ* 5.09 (1 H, br s)], one terminal methylene group at *δ* 4.84 (1 H, s) and 4.97 (1 H, s), two olefinic methylenes at *δ* 5.39 (1 H, d, *J* = 5.6 *Hz*) and *δ* 5.64 (1 H, br s)], together with signals from typical benzoyl group *δ* 8.18 (2 H, d, *J* = 7.2 *Hz*), 7.35 (2 H, d, *J* = 7.6 *Hz*), 7.46(1 H, t, *J* = 7.4 *Hz*)] (Table 1). ^13^ C-NMR and DEPT spectra of **1** showed signals from 38 carbons, including one carboxyl carbon *δ* 178.6 (C-21)], two carbons from terminal methylene group *δ* 107.0 (C-31) and 156.1 (C-24)], four olefinic carbons *δ* 116.7 (C-11), 120.8 (C-7), 142.9 (C-8) and 146.0 (C-9)], two oxygenated methylenes *δ* 79.0 (C-3) and 76.4 (C-16)], seven methyl carbons *δ* 17.6 (C-18), 21.9 (C-27), 22.0 (C-26), 22.4 (C-29), 22.7 (C-19), 26.6 (C-30) and 28.1 (C-28)], signals from benzoyl group *δ* 165.9 (C-7′), 133.2 (C-4′), 131.4 (C-1′), 129.8 (C-2′, 6′), and 128.9 (C-3′, 5′)], and signals from other fifteen carbons (see Table 2). The aforementioned NMR features were similar to those of 3-*epi*-dehydrotumulosic acid (**6**), except for the existence of an additional set of signals arising from the benzoyl group in **1**[13].

The downfield shift at C-3 (*δ* 79.0) in **1**, from (*δ* 75.1) in **6**, suggested that the additional benzoyl group was linked to C-3 position of dehydrotumulosic acid moiety. It was further confirmed by the HMBC experiment which showed correlation between H-3 (*δ* 5.09) with the signal from C-7′ (*δ* 165.9) of the benzoyl groups.

The relative configuration was established by ^1^ H-NMR and the NOESY experiment, in which the H-3 appeared as a broad singlet, the NOESY correlations of H-3β at (*δ* 5.09, 1 H, br s) with Me-29β at (*δ* 0.95, 3 H, s) revealed the benzoyl linked the α position of C-3 in compound **1**. On the basis of the above evidence, the structure of **1** was elucidated as 3α-benzoyl-16α-dihydroxyl-lanost-7, 9(11), 24(31)-trien-21-oic acid, named as 3-*epi*-benzoyloxyl-dehydrotumulosic acid.

Compound **2** was obtained as a colourless needle in CH_3_OH. Careful comparison of ^13^ C-NMR spectra of **1** and **2** indicate that both have a similar lanostane skeleton with different substitution group (details in Table 2). Unlike compound **1** with a benzoyl group, compound **2** showed signals from a malonyl group *δ* 41.9 (−CH_2_-), 166.4 (−COO-) and 167.6 (−COO-)] and a methoxyl group *δ* 52.2 (−OCH_3_)]. HMBC experiment showed correlations between methoxyl proton (*δ* 3.63) with 3′-C (*δ* 166.4, from malonyl group) indicated the methyl malonate group [14]. The HMBC experiment of **2** revealed the correlation between H-3 (*δ* 4.86) and C-1′ (*δ* 167.6), indicated the 3-substitution. Thus, compound **2** was established as 3-α-methyl-malonyl-16α-dihydroxy-lanost-7, 9(11), 24(31)-trien-21-oic acid, named as 3-*epi*-(3′-*O*-methyl malonyloxy)-dehydrotumulosic acid.

The ^13^ C-NMR spectra of **3** showed signals from a lanostane skeleton similar to those of 1 and 2 (Table 2), except with different substitution groups. Except signals from lanostane skeleton in compound **3**^1^ H-NMR showed signals at *δ* 3.12 (1 H, d, *J* = 15.2 *Hz*, H-2′), 3.16 (1 H, d, *J* = 15.2 *Hz*, H-2′), 3.02 (1 H, d, *J* = 14.4 *Hz*, H-4′), 3.08 (1 H, d, *J* = 14.4 *Hz*, H-4′) and 1.71 (3 H, s, -CH_3_)] along with ^13^ C-NMR showed signals *δ* 171.4 (C-1′), 46.3 (C-2′), 69.9 (C-3′), 46.4 (C-4′), 174.6 (C-5′), and 28.4 (−CH_3_)]. Those signals were assigned to 3-hydroxy-3-methylglutaryl group based on HMQC and HMBC spectra data. It was further confirmed from ESI-MS experiment, which showed fragment ions at *m/z* 525.4 [M-H-102 (CH (CH_3_) (OH)-CH_2_-COOH)]^-^. The HMBC correlations of H-3 (*δ* 4.94 br s) with C-1′ (*δ* 171.4) confirmed that the 3-hydroxy-3-methylglutaryloxyl group was at C-3 in **3** (Figure 1). The compound **3** is levorotatory. The *R*-configurations of C(3′) in **3** was deduced by comparing of the compound **3** specific rotation features with those of (+)-3-*epi*-dehydrotumulosic acid, and (3′ *S*)-(+)-3-hydroxy-3-methylglutaric acid, which are dextrorotatory [8,13]. These evidences indicated *R*-configuration of C (3′) in compound **3**. As stated above, the structure of **3** was indicated as 3-α-(3′-hydroxy-3′-methylglutaryloxy)-16α-dihydroxy-lanost-7, 9(11), 24(31)-trien-21-oic acid, named as 3-*epi*-(3′-hydroxy-3′-methylglutaryloxyl)-dehydrotumulosic acid.

## Experimental

### General experimental procedures

Optical rotations were measured on a P-1020 Polarimeter (JASCO, Tokyo, Japan). UV spectra were obtained on a UV 210A Shimadzu spectrometer. IR spectra were recorded on an FT-IR spectrometer (Nicolet iS10, Thermo Scientifi, USA) with KBr pellets. ^1^ H- and ^13^ C-NMR spectrum was recorded in pyridine-*d*_5_ with Bruker AM-400, DRX-500 and VARIAN INOVA-600 spectrometers operating at 400, 500 and 600 MHz for ^1^ H-NMR experiments, and 125 and 150 MHz for ^13^ C-NMR experiment, respectively. Coupling constants are expressed in Hertz (*Hz*) and chemical shifts are given on a *δ* (ppm) scale with tetramethylsilane as internal standard. Negative ion ESI-MS and HRESI-MS were recorded on an AutoSpec 3000 spectrometer (VG, Manchester, UK). Column chromatography separations were performed using HPD-826 (Cangzhou Bon Adsorber Technology Co., Cangzhou, China), Chromatorex ODS (Fuji Silysia Chemical Co., Greenville, USA) and Silica gel (Qingdao Haiyang Chemical Co., Qingdao, China) as adsorbants. TLC was carried on silica gel G precoated plates (Qingdao Haiyang Chemical Co., Qingdao, China). The TLC plate was monitored by spraying with 10% H_2_SO_4_ solution in ethanol followed by heating.

### Fungal material

The dried sclerotia of *W*. *extensa* were collected from Hebei Guang Ming Prepared Medicinal Herbs Co., Ltd, China and identified by Prof. Yu-Ting Cheng (Beijing University of Chinese Medicines). An authentic sample was kept in School of Chinese Pharmacy, Beijing University of Chinese Medicines.

### Extraction and isolation

The dried sclerotia of *W*. *extensa* (17.5 kg) were powdered and extracted with exhaustively 95% EtOH under reflux. The EtOH extract was concentrated to the small volume (3 L), and applied on a HPD-826 macroporous adsorptive resin (15 Kg, 18 cm × 150 cm), eluting with H_2_O (60 L) and 90% EtOH (80 L). The 90% EtOH fraction was concentrated under reduced pressure, and the residue (60 g) was subjected to column chromatography (CC) on silica gel eluted with CHCl_3_/CH_3_OH (4:1 to 1:1, 5 L) to obtain eight fractions (Fr 1–Fr 8). Fr 1, was further fractionated on silica gel eluted with cyclohexane/CHCl_3_ (8:1 and 4:1, each 1 L), and ODS eluted with a step gradient of H_2_O/MeOH (1:0 → 0:1), and PTLC (Cyclohexane/CHCl_3_/HOAc, 3:1:0.1) to give **1** (20 mg), **2** (10 mg) and **4** (10 mg). Fraction 2 was fractionated repeatedly on Silica gel (CHCl_3_/EtOAc, 8:1) and ODS (CH_3_OH/H_2_O, 75:25 → 85:15), eluted with CHCl_3_/CH_3_OH (50:1), to obtain **5** (20 mg) and **6** (10 mg) from Fr **2**. Fr **3** was subjected to CC on silica gel (CHCl_3_/EtOAc, 4:1), and preparative TLC on silica gel (CHCl_3_/EtOAc/HOAc, 1:1:0.1) to obtain **3** (20 mg).

#### *3-epi-benzoyloxyl-dehydrotumulosic acid (1)*

Colourless needles; ^1^ H-NMR (in pyridine-*d*_5_): see Table 1. ^13^ C-NMR (in pyridine-*d*_5_): see Table 2. IR (KBr) cm^-1^: 3400, 2928, 1710, 1641, 1279, 1175, 895, 800. UV λMeOH max nm (logϵ): 234 (4.32). HRESI-MS (*m/z*): 589.3864 [M + H]^+^, calcd for C_38_H_53_O_5_, 589.3893. ESI-MS (*m/z*) (rel. int.): 587.3 [M - 1]^-^ (100), 417.0 (23), 338.9 (4), 208.8 (13).

#### *3-epi-(3′-O-methyl malonyloxy)-dehydrotumulosic acid (2)*

Colourless needles; ^1^ H-NMR (in pyridine-*d*_5_): see Table 1. ^13^ C-NMR (in pyridine-*d*_5_): see Table 2. IR (KBr) cm^-1^: 3416, 2960, 1736, 1707, 1641, 1254, 1152, 891, 800. UV λMeOH max nm (logϵ): 243 (4.16). HRESI-MS (*m/z*): 607.3605 [M + Na]^+^, calcd for C_35_H_52_O_7_Na, 607.3611.

#### *3-epi-(3′ -hydroxy-3′-methylglutaryloxyl)-dehydrotumulosic acid (3)*

Colourless needles; [α] = − 7.6 (*c* = 0.1705, pyridine); ^1^ H-NMR (in pyridine-*d*_5_): see Table 1. ^13^ C-NMR (in pyridine-*d*_5_): see Table 2. IR (KBr) cm^-1^: 3389, 2962, 1707, 1642, 1205, 1176, 891, 802, 780, 770. UV λMeOH max nm (logϵ): 244 (4.13); HRESI-MS (*m/z*): 651.3880 [M + Na]^+^, calcd for C_37_H_56_O_8_Na, 651.3873. ESI-MS (*m/z*) (rel. int.): 627.5 [M - 1]^-^ (100), 525.4 (5).

## Competing interests

The authors declare that they have no competing interests.

## Authors’ contributions

GS carried out the chemical analysis-structure elucidation and drafted the Manuscript; NZ carried out the chemical studies; SW employed in the several chemical assays of extraction and isolation; YL worked at the part of experimental design; YB engaged in the part of chemical analysis-structure elucidation; CS carried out the part of chemical assays of extraction and isolation; SR conceived of the study and its design and coordination of the scientific teams. All authors have read and approved the final manuscript.
